# Molecular mechanisms mediating the G protein-coupled receptor regulation of cell cycle progression

**DOI:** 10.1186/1750-2187-2-2

**Published:** 2007-02-26

**Authors:** David C New, Yung H Wong

**Affiliations:** 1 Department of Biochemistry, the Molecular Neuroscience Center, and the Biotechnology Research Institute, Hong Kong University of Science and Technology, Clearwater Bay, Hong Kong, China

## Abstract

G protein-coupled receptors are key regulators of cellular communication, mediating the efficient coordination of a cell's responses to extracellular stimuli. When stimulated these receptors modulate the activity of a wide range of intracellular signalling pathways that facilitate the ordered development, growth and reproduction of the organism. There is now a growing body of evidence examining the mechanisms by which G protein-coupled receptors are able to regulate the expression, activity, localization and stability of cell cycle regulatory proteins that either promote or inhibit the initiation of DNA synthesis. In this review, we will detail the intracellular pathways that mediate the G protein-coupled receptor regulation of cellular proliferation, specifically the progression from the G1 phase to the S phase of the cell cycle.

## Background

An efficient system of cellular communication has evolved to ensure the ordered development, growth, maintenance and reproduction of multicellular organisms. This allows cells to respond to environmental stimuli as well as to each other by integrating the numerous extracellular and intercellular cues that they are constantly receiving into a coordinated response. Central to cellular signalling are the G protein-coupled receptors (GPCRs). The human genome is estimated to encode 800 to 1000 of these seven-transmembrane spanning proteins [1,2]. Activated GPCRs promote a wide spectrum of intracellular biochemical changes resulting in the modulation of many aspects of physiology, growth, development and disease control [3]. GPCRs have long been known to mediate mitogenic signals leading to cellular proliferation [4] and the overexpression or mutation of many GPCR subtypes in numerous cell types is thought to contribute to deregulated growth and tumour development [5,6].

Eukaryotic cell cycle progression is driven by a coordinated series of phosphorylation events, chiefly mediated by the cyclin-dependent kinase (CDK) family of serine/threonine kinases. The activity of the CDKs is, in turn, regulated by their phosphorylation status as well as by their interaction with numerous activating and inhibitory binding proteins. Active CDK complexes drive the cell cycle through its phases by phosphorylating downstream proteins [7]. During the G1 phase of the cell cycle, these CDK-driven events are responsive to extracellular cues. It is during this period of the cell cycle that GPCR-induced signal transduction pathways are able to affect, either negatively or positively, cell cycle progression. In this review we will examine the ability of GPCRs to modulate the activity of intracellular pathways that connect activation at the cell membrane to cellular proliferation.

### Heterotrimeric G proteins

GPCRs predominantly, although not exclusively [8], exert their effects by activating heterotrimeric G proteins. This promotes the release of free Gα and Gβγ subunits, which then initiate intracellular signal transduction. GPCRs preferentially couple to heterotrimeric G proteins that are grouped into four classes, known as Gα_q/11_, Gα_i/o_, Gα_s _and Gα_12/13 _[9]. Members of all four classes of Gα subunit have been shown to be involved in the regulation of cell growth and proliferation by virtue of the fact that constitutively active Gα mutants have been found in numerous tumours. The *gsp *oncogene (for G_s _protein) is a mutationally active form of Gα_s _detected in pituitary and thyroid tumours that promotes cell growth by constitutively activating adenylyl cyclase (AC). The *gip2 *oncogene (for G_i _protein) promotes tumour growth by activating mitogen-activated protein kinase (MAPK) pathways [10], while mutationally activated forms of Gα_z_, Gα_q_, Gα_12 _and Gα_13 _are able to generated transformed phenotypes [10,11].

Numerous GPCRs utilize heterotrimeric G proteins to modulate cellular proliferation. Direct evidence of the involvement of G_i/o _proteins has been obtained by the use of *pertussis *toxin (PTX) to block G_i/o_-mediated signalling. For example, melatonin acting on G_i/o_-coupled MT_1 _receptors expressed in MCF-7 breast cancer cells suppresses estrogen and glucocorticoid-induced cell proliferation [12], possibly by inhibiting the steroid receptor-induced transcription of the cyclin D1 gene [13,14]. These effects of melatonin are entirely blocked by PTX. The use of PTX has also indicated that G_i/o _proteins mediate the promotion of DNA synthesis by α_1_-adrenergic receptors in osteoblasts [15], κ-opioid receptors in C6 glioma cells [16] and lysophosphatidic acid (LPA) receptors in human fibroblasts [17]. Further examples of GPCR utilization of G_i/o _proteins in proliferative responses can be found in Table 1.

**Table 1:** GPCR-mediated activation of signalling pathways leading to cell cycle modulation

	**Intracellular Pathway**	**Cell Cycle Effect**	**References**
**GPCR**			
*G*_i/o_*-coupled*			
α_1_-adrenergic		↑DNA synthesis	[15]
	↑Src/C3G/Rap-1/B-Raf/ERK	↑Proliferation	[77]
Adenosine A_3_	↑PI3K/Akt/↓ERK	↓Proliferation	[98]
CXCR1/2	↑MMP/EGFR/ERK	↑Proliferation	[40]
CXCR3	↑ERK, ↑p38	↑DNA synthesis	[99]
CXCR4	↑Pyk2/PI3K/ERK	↑DNA synthesis	[71]
Dopamine D_2_	↑PKC/NF-κB	↑p21^Cip1^, ↑p27^Kip1^	[60]
	↑Src/C3G/Rap-1/B-Raf/ERK	↑Proliferation	[77]
Dopamine D_4_	↑Src/SHC/Ras/ERK	↑DNA synthesis	[78]
Sphingosine 1-phosphate EDG-1	↑p70^rsk^	↑Cyclin D1	[96]
	↑PDGFβ/ERK	↑Proliferation	[100]
κ-opioid	↑PLC/PKC/Ras/ERK	↑DNA synthesis	[16]
Lysophosphatidic acid LPA		↑DNA synthesis	[17]
Melatonin MT_1_	↓ERα/glucocorticoid receptor	↓Cyclin D1	[12, 13]
Serotonin 5HT_1E_	↑Src/C3G/Rap-1/B-Raf/ERK	↑Proliferation	[77]
Somatostatin SST_1/4/5_	↑ERK	↑p21^Cip1^, ↑p27^Kip1^	[50]
Somatostatin SST_2_	↑PI3K/Ras/Rap-1/B-Raf/ERK	↑p27^Kip1^	[90]
Somatostatin SST_2a_	↑p38	↑p21^Cip1^	[91]
Somatostatin SST_2b_	↑PI3K/p70^rsk^/Akt	↑Proliferation	[91]
*G*_s_*-coupled*			
Dopamine D_1_	↑PLCβ/↓Raf-1	↓Cyclin D1/↑p27^Kip1^	[101]
Glucagon-like peptide GLP-1	EGFR/PI3K	↑Proliferation	[42]
Glucagon-like peptide GLP-2		↑DNA synthesis	[18]
GPR30	↑PKA/CREB	↑Cyclin D2/CDK4-6 complex formation	[27, 28]
Lysophosphatidic acid LPA		↑Proliferation	[19]
Melanocortin MC_5_	↑JAK/STAT	↑Proliferation	[82]
Parathyroid PTH	↑cAMP/PKA	↑p27^Kip1^	[7, 22]
	↑cAMP/Epac/Rap-1/B-Raf/ERK	↑Proliferation	[51]
	↑cAMP/↑PKA/↓Raf-1	↓Proliferation	[51]
	↑MKP-1/↓ERK	↓Cyclin D1, ↑p21^Cip1^	[52]
Thyroid stimulating hormone TSH	↑cAMP/CREB/c-Fos	↑DNA synthesis, ↑Cyclins D1/E	[14, 23-25]
	↑PKA/Ras/PI3K	↑DNA synthesis	[102]
*G*_q_*-coupled*			
α_1B_-adrenergic	↑PKC/Raf-1/ERK	↑Proliferation	[34]
	↑JNK, ↑p38	↓Proliferation	[55]
	↑Src/Dbs/cdc42/MKK4/JNK	↓Proliferation	[76]
	↑Ras/Rac/JAK/STAT	↑Proliferation	[81]
Angiotensin II	↑MMP/EGFR/ERK	↑Cyclin D1	[39]
	↑Ras/ERK/c-Fos/c-Jun	↑Cyclin D1, ↑pRB phosphorylation	[48]
	↑p125FAK/Rac1/JNK	↑Proliferation	[67]
Bombesin	↑MMP/EGFR/PI3K	↑Cyclins D1/E	[41]
	↑PKD	↑Proliferation	[58]
Bradykinin	↑MMP/EGFR/PI3K	↑Cyclins D1/E	[41]
Endothelin	↑MMP/EGFR/ERK	↑DNA synthesis	[39]
	↑PLCβ/Ca^2+^/Src/ERK	↑Proliferation	[74]
	↑Src/Rho/p125FAK/paxillin	↑DNA synthesis	[70]
	↑Pyk2/ERK	↑DNA synthesis	[70]
Gastrin-activated CCK2	↑Rho/integrin/p125FAK/paxillin	↑Proliferation	[68,69]
	↑PKC/Src/p38	↑Proliferation	[75]
	↑JAK/STAT	↑Proliferation	[80]
Lysophosphatidic acid LPA	↑MMP/EGFR/ERK	↑cyclin D1	[39]
Muscarinic M_1_	↑PKC/Raf-1/ERK	↑Proliferation	[34]
Muscarinic M_3_	↑JNK/c-Jun/SP-1	↓DNA synthesis, ↑p21^Cip1^/CDK2, ↓pRb phosphorylation	[56]
Muscarinic M_5_	↑Ras/Rac/JAK/STAT	↑Proliferation	[81]
Muscarinic subtypes	↑Src/ERK/CREB	↑DNA synthesis	[103]
Platelet-Activating Factor receptor	↑MMP/EGFR/ERK	↑Proliferation	[104]
Purinergic P2Y_2/4_	↑PKC/Raf/MAPK	↑DNA synthesis	[49]
Substance P (NK-1)	↑Src/PKCδ/ERK	↑Proliferation	[72]
Thrombin	↑MMP/EGFR/ERK	↑DNA synthesis	[39]
	↑RhoA/PI3K/Akt	↓p27^Kip1^, ↑Cyclin D1/CDK4	[92-94]
	↑ERK	↑CDK2 nuclear translocation	[95]
	↑PI3K/Akt,		
Vasopressin V_1A_	↑PKD	↑Proliferation	[58]
	↑Ca^2+^/PI3K/PKC/ERK	↑G1-S phase	[105]
	↑EGFR/Pyk2/Src/ERK/PI3K	↑Proliferation	[106]
*G*_12/13_*-coupled*			
Lysophosphatidic acid LPA		↑DNA synthesis, ↑Proliferation	[20]
	↑EGFR/Rho/ROCK	↑Cyclins A/D1, ↑p21^Cip1^, ↓p27^Kip1^	[43,45]
	↑JNK	↑Cyclin A	[20,54]

A selection of examples is presented that demonstrate the involvement of GPCR-mediated intracellular signalling pathways in the regulation of cell cycle progression. ↑, indicates an increase in protein levels or activity; ↓, indicates a decrease in protein levels or activity.

The involvement of G_s _proteins in a few GPCR-initiated responses has been determined using *cholera *toxin (CTX), which constitutively activates Gα_s _subunits, preventing further activation by GPCRs. Glucagon-like peptide 2 (GLP-2) acts as a potent mitogen at Caco-2 intestinal epithelial cells but pretreatment of cells with CTX significantly reduces GLP-2-induced DNA synthesis [18]. Likewise, CTX blocks the LPA-induced proliferation of retinal pigment epithelial cells [19], although the relative contribution of LPA receptor activation of G_i/o _and G_s _proteins in these responses was not determined. Other G_s_-coupled GPCRs also play significant roles in promoting or inhibiting cell cycle progression, as witnessed by their effects on downstream effectors (see Table 1 and below).

While there is much compelling evidence that proves the involvement of G_q/11 _and G_12/13_-activated signalling pathways in cell cycle control (discussed in more detail below), direct experimental evidence of the GPCR activation of these G proteins for the purposes of cell cycle control is generally absent. A notable exception, however, is a study of NIH3T3 fibroblasts transfected with Gα_12_. In the presence of LPA, these cells synthesize DNA and proliferate much more rapidly than untransfected cells, indicating that the LPA effects are mediated by the LPA receptor coupled to Gα_12 _[20].

### cAMP/PKA/CREB

**Figure 1:**
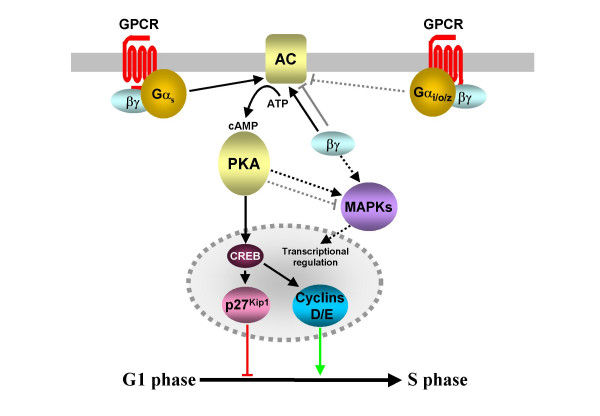
**Modulation of intracellular cAMP levels by GPCR-coupled mechanisms affects cell cycle progression**. Agonist activation of G_s_-coupled receptors promotes increased AC activity and cAMP accumulation. Subsequent PKA activation leads to the activation of the transcription factor CREB and the regulation of the expression of cyclins and the CDK inhibitor p27^Kip1^. The resulting effect on cell cycle progression is dependent on a number of factors, including the concentration of cAMP generated. PKA can also regulate, positively or negatively, other mitogenic pathways, particularly those leading to the activation of MAPKs, (see text for further details). Activation of the AC/cAMP/PKA axis can be antagonized by the activation of GPCRs coupled to G_i/o_-family proteins. However, the definitive involvement of these MAPK and G_i/o_-coupled pathways in regulating proliferation has not been established (indicated by dashed lines).

Cyclic AMP (cAMP) is generated from ATP by the AC family of enzymes. ACs are activated by Gα_s _subunits while most isoforms are inhibited by Gα_i/o _subunits. Gβγ dimers can either negatively or positively regulate AC isoforms. cAMP activates protein kinase A (PKA), which not only phosphorylates transcription factors, including the cAMP response element binding protein (CREB) and AP1 family members, but also modulates the activity of other signalling pathways (Fig. 1 and [21]).

Parathyroid hormone (PTH) receptor activation in UMR-106 osteoblast cells inhibits the progression of cells into S phase. This blockage is accompanied by increases in p27^Kip1^, an inhibitor of the cyclin-CDK complexes necessary for the G1 to S phase transition [7]. As PTH is a G_s_-coupled receptor, a cell permeable cAMP analogue mimicked the effects of PTH while a PKA inhibitor abolished the increases in p27^Kip1 ^levels [22]. In complete contrast, activation of the thyroid stimulating hormone (TSH) receptor, also G_s_-coupled, induced G1 to S phase progression in rat thyroid cells. The TSH-induced progression and increased DNA synthesis was associated with increases in the levels of c-Fos [23], a binding site for which is found in the promoter region of cyclin D1 [14], as well as increases in the levels of two G1 cyclins, D1 and E [24]. These effects were mimicked by a cAMP analogue [24] and cells containing a dominant negative mutant of CREB, which also activates the cyclin D1 promoter, had reduced levels of TSH-induced DNA synthesis and an increased cell cycle length [25]. Similarly, estrogen and 17β-estradiol (E2) are thought to act, in part, as ligands for the orphan GPCR GPR30 [26]. The E2-induced proliferation of keratinocytes is accompanied by increases in the levels of cyclin D2, a key mediator of G1 to S phase progression in skin cells [27], and increases in the activity of cyclin D2-CDK4 or 6 complexes [28]. E2 increased the amount of active CREB, a transcriptional activator of the cyclin D2 gene, and this, as well as the increased levels of cyclin D2 and proliferation, were reversed by a PKA inhibitor [28].

Due to the differential expression patterns and levels of AC isoforms, the multiplicity of phosphodiesterases that can degrade cAMP and the regulation of ACs by Ca^2+^/calmodulin and a variety of kinases [21], it is perhaps not surprising that activation of G_s_-coupled receptors can lead to contradictory effects on cell cycle progression depending on the cell type and GPCR studied (Table 1 and [14]). It has been suggested that the differences may be the result of different cAMP concentrations, with lower levels inducing cyclin D expression whereas higher levels induce p27^Kip1 ^expression [28]. In addition, elevated levels of cAMP and the activation of PKA results in cell type specific modulation of MAPK pathways [29], while it is probable that Gβγ subunits released from GPCR-activated G_s _proteins can activate MAPKs (Fig. 1 and [30]).

It is not yet clear whether G_i/o_-coupled GPCR-induced reductions in basal cAMP levels can independently affect cell cycle progression but it is likely that intracellular cAMP levels are the product of competing signals from G_s _and G_i/o _proteins. There are examples of G_i/o_-coupled receptors modulating cell cycle progression, e.g. the melatonin MT_1 _receptor-mediated inhibition of proliferation in rat uterine cells [31], however these effects are likely to be mediated by a variety of other intracellular pathways (see following sections) rather than by the inhibition of AC activity.

### MAPK pathways

Mammalian cells express three major classes of MAPKs, the extracellular signal-regulated kinases (ERK), c-Jun N-terminal kinase/stress-activated protein kinases (JNK/SAPK) and p38 kinases, the activation of which results in the stimulation of transcription factors and the regulation of the expression of cell cycle proteins [32,33]. GPCRs activate MAPKs via several distinct mechanisms, *i.e. *by using β-arrestin/endocytotic pathways, transactivating RTKs or by second messenger activation. The β-arrestin pathway generally results in the retention of MAPKs in the cytoplasm and transient MAPK activity, limiting their role in the activation of nuclear substrates and proliferation (discussed in [34]). However, GPCR activation of β-arrestin dependent pathways does not exclude the possibility of sustained ERK activation [35] or of nuclear translocation of ERK activity and the promotion of proliferation, as demonstrated for the neurokinin NK-1 receptor [36]. In contrast, RTK-mediated and second messenger activation of MAPK pathways generate the sustained MAPK activity that is often thought critical to the GPCR regulation of cell cycle progression [32].

#### RTK transactivation

**Figure 2:**
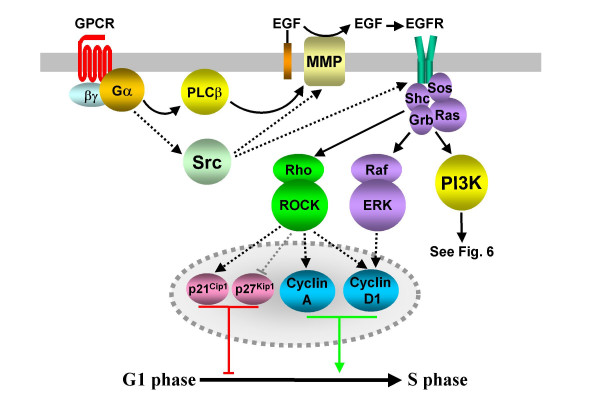
**GPCR transactivation of EGFR leads to the activation of multiple mitogenic pathways**. GPCR/G protein activity of many families of G protein promotes the activity of MMPs via PLCβ-dependent, or possibly Src-dependent (indicated by dashed lines – see text for further details), mechanisms. MMP activity releases EGF in its soluble form. The resulting EGFR activity promotes the formation of a signalling complex and the activation of PI3K, MAPK and ROCK kinases in a GPCR and cell type specific manner. The increased expression of cyclins promotes progression into S phase, while the upregulation of CDK inhibitors p21^Cip1 ^and p27^Kip1 ^delays S phase entry. Dashed lines also identify the probable involvement of multiple, unidentified intermediates in the transcriptional regulation of cell cycle proteins.

It is often observed that GPCR-mediated proliferation is the result of the Gα or Gβγ subunit transactivation of RTKs [37,38]. Ligands for the LPA, endothelin-1 and thrombin receptors all promote S phase entry and DNA synthesis in Rat-1 fibroblasts by transactivating the epidermal growth factor receptor (EGFR, an RTK). Such transactivation requires the activation of matrix metalloproteases (MMPs) to release EGF from its membrane bound form, which then stimulates the EGFR and downstream ERK pathways (Fig. 2 and [39]). The same study also demonstrated that LPA and angiotensin II promoted cyclin D1 accumulation in the G1 phase of kidney cancer cells via the same MMP/EGFR/ERK pathway [39], while a similar proliferative pathway is activated by G_i/o_-coupled CXCR1/2 receptors in Caco-2 cells [40]. However, in Swiss 3T3 cells bradykinin and bombesin promote cyclin D1 and E expression in mid to late G1 in an EGFR-dependent but ERK pathway-independent manner [41]. This ERK-independent pathway may involve the RTK activation of phosphotidylinositol 3-kinase (PI3K)/Akt cascades (see below and Figs. 2 and 6), as might the G_s_-coupled GLP-1 receptor promotion of proliferation in β-cells [42].

As for receptors acting via G_12/13 _heterotrimers, LPA receptors stimulate Rho, a member of the Ras superfamily [43], and its effector Rho kinase (ROCK; [44]) utilizing EGFRs. This potentially leads to the stimulation of several signal transduction pathways and the regulation of the levels of cyclins A and D1 as well as the CDK inhibitors p21^Cip1 ^and p27^Kip1 ^(Fig. 2 and [45]).

A number of other proliferation-inducing RTKs are also transactivated by GPCRs (reviewed in [46]). It is not yet clear whether activation of these RTKs requires GPCR-induced cleavage of membrane-bound RTK ligands by MMPs or whether this requirement can be bypassed by the GPCR-induced Src family tyrosine kinase activation of RTKs (Fig. 2 and [46]). It is also yet to be determined what role GPCR/EGFR activation of JNK and p38 play in proliferative responses [38]. It has, however, been reported that G_i/o_-coupled GPCR-induced JNK activity can be synergistically increased upon EGF co-stimulation, although this may not necessarily require transactivation [47].

#### Second messengers

**Figure 3:**
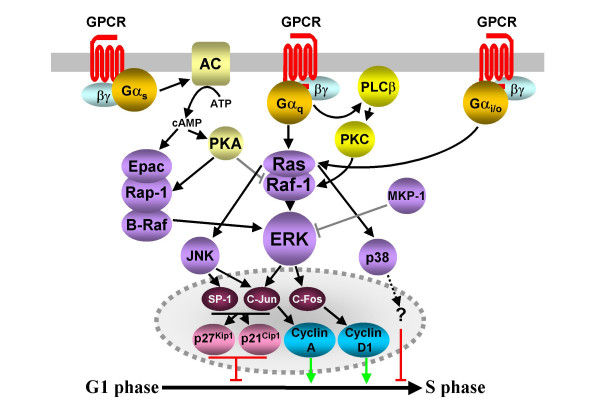
**GPCR-mediated activation of MAPKs is also regulated by the generation of intracellular messengers**. GPCR activity leads to the activation of AC/cAMP and PLCβ/PKC second messenger pathways. cAMP directly, or via PKA, activates RAP-1/B-Raf/ERK pathways, and potentially inhibits Raf-1 activated ERK activity. The Gα_q_/PLCβ/PKC pathway promotes Ras/Raf-1/ERK activity, and it is likely that G_q_- and G_i/o_-coupled GPCRs can activate JNKs and p38. The result of the interplay between these pathways is either proliferative or antiproliferative, depending on the expression of GPCRs and signalling intermediates. Dashed indicators identify the probable involvement of multiple, unidentified intermediates.

GPCRs can also promote the MAPK-dependent transcription of cell cycle proteins without transactivating RTKs [33]. Mitogenic pathways activated by different Gα families have been described in detail. Angiotensin II promotes DNA synthesis and proliferation in many cell types by activating the G_q_-coupled AT_1 _receptor. AT_1 _receptor activity in human adrenal cells induces Ras-dependent ERK activity, leading to increased levels of c-Fos and c-Jun transcription factors and increases in cyclin D1 promoter activity, cyclin D1 protein levels and pRB hyperphosphorylation (Fig. 3 and [48]). Other mitogenic GPCRs, including M_1 _muscarinic and α_1B_-adrenergic and purinergic receptors, induce ERK activity via the Ras-independent PKC phosphorylation and activation of Raf-1 [34,49]. However, there are reports of GPCRs using seemingly similar ERK pathways to promote G1 phase arrest. For example, several of the G_i/o_-coupled somatostatin receptors inhibit cell cycle progression in a variety of cell types by promoting accumulation of the CDK inhibitors p27^Kip1 ^and p21^Cip1 ^(Fig. 3 and [50]).

G_s_-coupled GPCRs utilize the Epac/Rap-1/B-Raf pathway to activate MAPK cascades and proliferation. In bone cells expressing B-Raf, PTH promotes cAMP accumulation, which binds directly to the Rap-1 guanine nucleotide exchange factor Epac. Epac in turn activates Rap-1, a Ras family GTPase, which activates the kinase B-Raf, triggering ERK cascades [51]. Alternatively, PKA may directly activate Rap-1 (Fig. 3 and [34]). Interestingly, it now seems clear that in cells lacking B-Raf, GPCR-mediated activation of AC leads to the PKA phosphorylation and inhibition of Raf-1 [34], and/or the antagonism of the Ras activation of Raf-1 by Rap-1 [51]. Therefore, in cells with reduced levels of B-Raf, G_s_-coupled receptor activation leads to the inhibition of the canonical Ras/Raf/ERK mitogenic pathway. This inhibition may be reinforced by the induction of MAPK phosphatase-1 (MKP-1), which dephosphorylates and inactivates ERKs. In bone cells this may account for the PTH-induced inhibition of the ERK-mediated expression of cyclin D1, arresting cells in G1 phase [52]. The ability of G_i/o_-coupled receptors to utilize Rap-1/B-Raf pathways to modulate proliferation is not yet clear but the potential for such a pathway to operate is apparent as dopamine D_2 _receptors are able to use G_o _proteins as intermediaries to activate B-Raf [53].

The JNK and p38 kinases do not seem to be as commonly involved in the transduction of GPCR-induced proliferative signals, yet JNKs do mediate the LPA-induced proliferation of NIH3T3 cells transfected with Gα_12 _[20], possibly via the induction of cyclin A at the G1-S phase transition [54]. In fact, JNKs and p38 kinases seem adept at mediating antiproliferative signals. In HEK293 cells, α_1B_-adrenergic receptor stimulation inhibited cell proliferation in a JNK- and p38-dependent manner [55]. In Chinese hamster ovary cells, activation of the G_q_-coupled muscarinic M_3 _receptors caused a G1 phase arrest and inhibited DNA synthesis by increasing the expression levels p21^Cip1^. The p21^Cip1 ^increased its association with CDK2, leading to an accumulation of hypophosphorylated pRB. M_3 _receptor activation promoted the activation of JNK and the phosphorylation of c-Jun. This enhanced the interaction of c-Jun with its transcriptional partner SP-1, possibly contributing to the enhancement of p21^Cip1 ^promoter activity (Fig. 3 and [56]).

### Other PKC-dependent pathways

**Figure 4:**
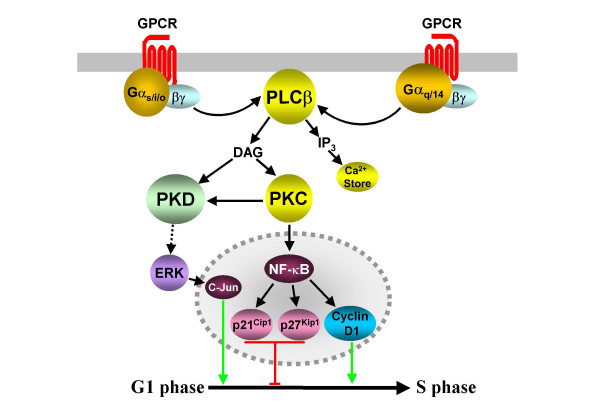
**Further PKC-dependent cell cycle regulation**. G_i/o_-, G_s_- and G_q_-family coupled GPCRs can activate PLCβ and PKC activity via Gα or Gβγ subunits. Activated PKC can phosphorylate and activate PKD, leading to the activity of ERK-dependent proliferative pathways. PKC is also able to initiate a series of events that promotes the transcriptional activity of NF-κB. NF-κB activates the promoter regions of cyclin D1 as well as those of p21^Cip1 ^and p27^Kip1^, causing S phase entry or delay. Dashed indicators identify the probable involvement of multiple, unidentified intermediates.

As well as its documented role in activating Raf-1 (see above), PKC also acts as a key mediator of a number of other GPCR-induced proliferative pathways. PKC isoforms, as well as DAG, are able to activate the protein kinase D (PKD) family of serine/threonine kinases [57]. Indeed, the proliferation of Swiss 3T3 cells in response to the activation of G_q_-coupled bombesin or vasopressin receptors is greatly potentiated by the overexpression of PKD [58]. The pathways connecting GPCR activation to the control of cell cycle progression have not yet been outlined but it is known that PKD can activate ERK pathways and phosphorylate c-Jun (Fig. 4 and [57]).

PKC also activates the NF-κB transcription factors by initiating a series of phosphorylation and degradation events [59]. In mouse embryonic cell lines expressing both dopamine D_1 _(G_s_-coupled) and D_2 _(G_i/o_-coupled) receptors, the administration of dopamine resulted in a PKC-dependent increase in NF-κB DNA binding activity, along with increases in the levels of p21^Cip1 ^and p27^Kip1 ^and an inhibition of DNA synthesis [60]. However, in an embryonic fibroblast model NF-κB binds to and activates the cyclin D1 promoter region, leading to G1 to S phase progression (Fig. 4 and [61]). Other GPCRs, including the G_i/o_-coupled μ-opioid receptor [62], the somatostatin SST_2 _receptor acting via Gα_14 _[63] and the adenosine A_1 _receptor acting via Gα_16 _[64] also promote NF-κB activation. This activity appears to be mediated by numerous intracellular pathways, including those dependent on PKC, ERK, Src, PI3K, JNK, and PLCβ, although the role of G_i/o_-coupled receptor activation of these pathways in NF-κB mediated cell cycle progression is yet to be investigated.

### Src family tyrosine kinases

**Figure 5:**
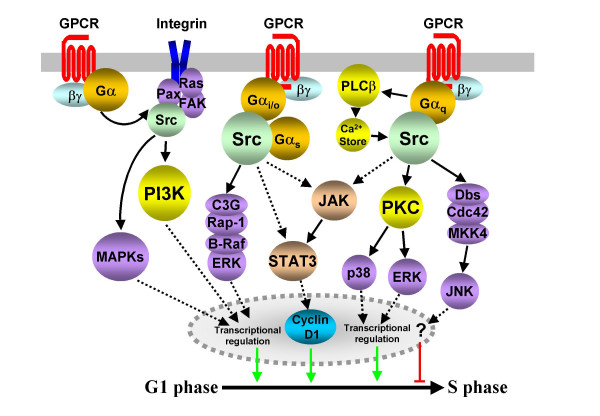
**Src family kinase-dependent cell cycle control**. G_q_-, G_12/13_- and G_i/o_-coupled GPCRs are all known to regulate mitogenesis via the transactivation of Src-dependent integrin signalling complexes. G_q_- and G_i/o_-coupled receptors also utilize Src to activate a variety of MAPK pathways. G_s_-, G_i/o_- and G_q_-coupled receptors promote proliferation via the activation of the STAT transcription factors, and this has been postulated to be Src-dependent (shown in dashed lines). Full STAT activity may require phosphorylation by JAKs and MAPKs. Dashed lines also identify the probable involvement of multiple, unidentified intermediates in the transcriptional regulation of cell cycle proteins.

Members of this family of kinases are firmly embedded in signal transduction pathways activated by diverse extracellular stimuli [65]. They also play a significant role in the crosstalk between many pathways. We have already seen that Src kinases play a part in the GPCR-induced transactivation of RTKs (see preceeding discussion and Fig. 2). The GPCR/Src/RTK sequence of events is poorly understood, involving either Gα or Gβγ subunit stimulation of Src or Src-activating pathways [46]. GPCRs can also transactivate focal adhesion complexes consisting of integrin heterodimers that act as extracellular matrix receptors. The transactivation is Src-dependent and leads to the formation of a signalling platform that includes Src, the focal adhesion kinase p125FAK or its homologue Pyk2, paxillin, as well as the adaptor proteins required to promote Ras family-dependent signalling pathways, particularly those that use MAPKs and PI3Ks as intermediates (reviewed in [66]). Angiotensin II utilizes just such a p125FAK/Rac1/JNK pathway to promote the proliferation of vascular smooth muscle cells [67]. Gastrin and other neuropeptides, through their agonistic effect on G_q_- and G_12/13_-coupled GPCRs, are also thought to promote G1 to S phase transition, in part, via their activation of similar Rho/integrin/p125FAK/paxillin signalling complexes [68,69]. This would include the endothelin receptors, which promote DNA synthesis in primary astrocytes using a combination of an adhesion dependent Src/Rho/p125FAK/paxillin and an apparently Rho/adhesion-independent Pyk2/ERK pathway [70]. The G_i/o_-coupled CXCR4 receptor promotes DNA synthesis via a Pyk2/PI3K/ERK pathway (Fig. 5 and [71]).

**Figure 6:**
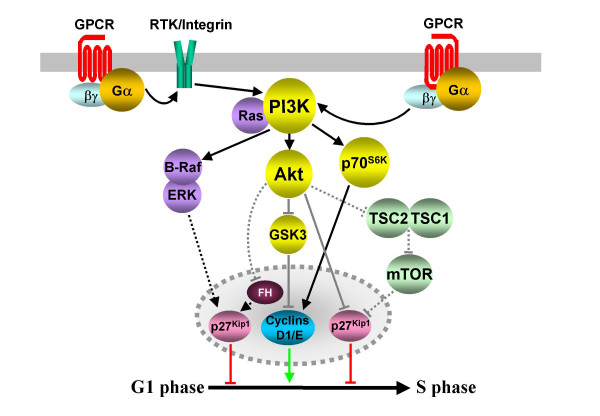
**Activation of PI3K-dependent cell cycle regulation**. The expression, stability and activity of cyclins and CDK inhibitors are regulated by the activity of several PI3K-dependent pathways. Numerous GPCRs activate PI3K isoforms either through Gβγ subunits or via RTK and integrin transactivation. PI3Ks activate ERKs and Akt, leading to the transcriptional regulation of p27^Kip1^. In addition, Akt phosphorylates p27^Kip1^, thereby affecting its nuclear localization. Acting through TSC1, TSC2 and mTOR, Akt can negatively affect the stability of p27^Kip1^, although GPCR regulation of proliferation through mTOR has not been established (indicated by dashed lines). PI3Ks may also promote proliferation by promoting cyclin expression (via p70^S6K^) and stability (via Akt and GSK3). Dashed lines also identify the probable involvement of multiple, unidentified intermediates in the transcriptional regulation of cell cycle proteins.

In the absence of RTK transactivation, Src activity is required for GPCR-induced proliferation of a number of alternative pathways. The G_q_-coupled substance P receptor (NK-1) promotes the proliferation of human glioblastoma cells in a Src-dependent manner. Inhibition of Src activity prevents the phosphorylation and activation of PKCδ and ERK in these cells [72]. ERKs are known substrates of PKCδ [73]. The mitogenic G_q_-coupled endothelin receptors activate ERKs via a Src-dependent pathway that requires the Gα_q_-subunit activation of PLCβ and Ca^2+ ^release [74]. A similar pathway was identified in CHO cells expressing the gastrin-activated CCK2 receptor, where proliferation was mediated by a PKC/Src activation of p38 MAPK [75]. In contrast, the anti-proliferative effects of the α_1B_-adrenergic receptor in HEK293 cells are Src family kinase dependent. Such activity stimulates a Rho family GEF, Dbs, and cdc42, a Rho family member, activating a MAPK kinase, MKK4, and JNK (Fig. 5 and [76]).

Other studies have shed light on the G_i/o_-coupled GPCR activation of Src-mediated proliferation. Serotonin 5HT_1E_, dopamine D_2 _and α_2C_-adrenergic receptors all promote the proliferation of NIH3T3 cells via the Gα_i_-subunit activation of Src, which activates C3G, a RapGEF. As was discussed above, RapGEFs, including Epac, activate Rap-1/B-Raf/ERK pathways leading to proliferation (Fig. 5 and [77]). Alternatively, the dopamine D_4 _receptor promotes DNA synthesis via Src/Src homology 2-containing protein (SHC)/Ras/ERK pathway [78]. The precise mechanism of Gα_i _activation of Src is still under investigation but both Gα_i _and Gα_s _directly bind to and activate Src family kinases [79].

Activation of MAPKs is not the only consequence of the GPCR-induced activation of Src family kinases. An increasing number of GPCRs activate the Janus kinase/signal transducer and activator of transcription (JAK/STAT) pathways as a means to modulate cell cycle progression. The gastrin-activated CCK2, muscarinic M_5 _and α_1B_-adrenergic G_q_-coupled receptors, as well as the G_s_-coupled melanocortin MC_5 _receptor induce increases in cell proliferation by activating JAK and STAT family members [80-82]. The definitive involvement of Src in these pathways has not been established and it is possible that a combination of the direct activation by Src kinases and Ras-dependent MAPK pathways is required for full STAT transcriptional activation [83]. Interestingly, the promiscuously coupled Gα_14 _and Gα_16 _subunits are similarly able to mediate the activation of Src and JAK/STAT pathways following activation of several GPCRs [84-86], although whether this leads to the modulation of cell cycle progression is not yet known. The ability of Gα_i/o _subunits to promote the Src-mediated activation of STATs is well documented [83]. What is less clear is the role of G_i/o_-coupled GPCRs in controlling cell cycle progression via these pathways. Intriguingly, in NIH3T3 cells, Gα_i2 _mediates the Src activation of STAT3, and this may promote the expression of cyclin D1 (Fig. 5 and [87]).

### PI3K/Akt pathways

Extracellular signals transduced by both RTKs and GPCRs converge upon the activation of a family of PI3Ks. Activation of these lipid kinases by GPCRs is thought to be dependent on the direct binding of Gβγ subunits and Ras to PI3Ks [88]. PI3K activation initiates a phosphorylation cascade leading to the activation of Akt (also termed protein kinase B) and its downstream kinases phosphoinositide-dependant kinase 1 (PDK1), glycogen synthase kinase 3 (GSK3), p70 ribosomal protein S6 kinase (p70^S6K^), mammalian target of rapamycin (mTOR) and others [89]. In addition, we have already seen how GPCRs can activate PI3K pathways via RTK or integrin transactivation [41,42,66]. Following direct or indirect GPCR-induced PI3K activation, cell cycle progression is regulated by the effect of PI3K-activated kinases on the expression and stability of cell cycle proteins, or by the modulation of the activity of other signal transduction pathways. For example, somatostatin SST_2 _receptors expressed in Chinese hamster ovary cells (CHO) inhibit proliferation by activating a PI3K-dependent Ras-Rap1/B-Raf/ERK pathway, resulting in an increase in the levels of p27^Kip1 ^protein (Fig. 6 and [90]). It has also been shown that sustained activation of p38 by activation of the SST_2a _receptor subtype leads to upregulation of p21^Cip1 ^and cell cycle inhibition. However, this can be antagonized by activation of SST_2b _receptor, which activates PI3K, p70^S6K^, Akt and proliferation (Fig. 6 and [91]). This suggests that the final outcome of a signalling event relies on the balance of several competing mechanisms.

Several studies have shed further light on the effect of the activation of GPCR/PI3K pathways on cell cycle proteins. For example, thrombin receptor activation in vascular smooth muscle cells leads to reduced levels of p27^Kip1 ^and increased cellular proliferation [92], while in embryonic fibroblasts the evidence suggests that thrombin receptor activation of PI3K/Akt pathways promotes cyclin D1 accumulation, cyclin D1-CDK4 activity and cell cycle progression [93,94]. Furthermore, it has been postulated that thrombin receptor activation of ERK activity ultimately leads to enhanced translocation of CDK2 into the nucleus and fibroblast proliferation [95]. Moreover, sphingosine 1-phosphate activation of the EDG-1 receptor activates p70^S6K^, promoting cyclin D1 expression and proliferation (Fig. 6 and [96]). The reduction in p27^Kip1 ^levels and the upregulation of cyclin D protein are thought to be the primary cell cycle effects of PI3K activation by RTKs [89]. The cyclin D1 protein is stabilized by the Akt-mediated inactivation of GSK3, which normally phosphorylates and promotes the degradation of cyclin D1. Akt also phosphorylates and inactivates forkhead (FH) transcription factors, which bind to and activate the p27^Kip1 ^promoter. PI3K pathways may also reduce the stability of p27^Kip1^, and Akt phosphorylation of p27^Kip1 ^adversely affects its nuclear localization. Akt-induced phosphorylation of the tumour suppressor TSC2 (also known as tuberin) causes the dissociation of TSC2 and TSC1 (also known as hamartin), relieving their inhibition of mTOR kinase. Increased mTOR activity reduces the stability of p27^Kip1 ^(Fig. 6 and [89]). Some GPCRs have now been shown to couple to this PI3K/tuberin system [97], although the significance for cellular proliferation has not been established.

## Conclusion

It is a common finding that GPCRs regulate cell cycle progression. The final effect on cellular proliferation is likely to be the result of the combined action of different GPCRs simultaneously activating several different G protein families, each of which affects the activity of multiple intracellular signalling pathways that modulate the expression, activity and stability of key proteins of the cell cycle machinery. Restrictions on GPCR-induced effects may arise from factors such as the expression and accessibility of signalling components as well as the magnitude and duration of the intracellular response. Yet to be studied in depth is the combined effect of GPCR activation along side the mitogenic effects of other classes of signalling molecules. Nevertheless, there is much hope that the targeted modulation of GPCR activity will reveal strategies for the treatment of medical conditions that arise due to deregulated cell growth and proliferation.

## Competing interests

The author(s) declare that they have no competing interests.

## Authors' contributions

YHW conceived of the review and revised it critically for important intellectual content. DCN drafted the manuscript.
